# Investigation of copy number variations on chromosome 21 detected by comparative genomic hybridization (CGH) microarray in patients with congenital anomalies

**DOI:** 10.1186/s13039-018-0391-3

**Published:** 2018-08-10

**Authors:** Wenfu Li, Xianfu Wang, Shibo Li

**Affiliations:** Genetics Laboratory, University of Oklahoma Health Sciences Center, 1122 NE 13th Street, Suite 1400, Oklahoma City, OK 73104 USA

**Keywords:** Chromosome 21, Microarray, CNV, Genotype-phenotype association, *CXADR*, *DYRK1A*

## Abstract

**Background:**

The clinical features of Down syndrome vary among individuals, with those most common being congenital heart disease, intellectual disability, developmental abnormity and dysmorphic features. Complex combination of Down syndrome phenotype could be produced by partially copy number variations (CNVs) on chromosome 21 as well. By comparing individual with partial CNVs of chromosome 21 with other patients of known CNVs and clinical phenotypes, we hope to provide a better understanding of the genotype-phenotype correlation of chromosome 21.

**Methods:**

A total of 2768 pediatric patients sample collected at the Genetics Laboratory at Oklahoma University Health Science Center were screened using CGH Microarray for CNVs on chromosome 21.

**Results:**

We report comprehensive clinical and molecular descriptions of six patients with microduplication and seven patients with microdeletion on the long arm of chromosome 21. Patients with microduplication have varied clinical features including developmental delay, microcephaly, facial dysmorphic features, pulmonary stenosis, autism, preauricular skin tag, eye pterygium, speech delay and pain insensitivity. We found that patients with microdeletion presented with developmental delay, microcephaly, intrauterine fetal demise, epilepsia partialis continua, congenital coronary anomaly and seizures.

**Conclusion:**

Three patients from our study combine with four patients in public database suggests an association between 21q21.1 microduplication of CXADR gene and patients with developmental delay. One patient with 21q22.13 microdeletion of DYRK1A shows association with microcephaly and scoliosis. Our findings helped pinpoint critical genes in the genotype-phenotype association with a high resolution of 0.1 Mb and expanded the clinical features observed in patients with CNVs on the long arm of chromosome 21.

**Electronic supplementary material:**

The online version of this article (10.1186/s13039-018-0391-3) contains supplementary material, which is available to authorized users.

## Background

Down Syndrome (DS) is the most prevalent genetic disorder resulting in intellectual disability, which is usually caused by an extra copy of chromosome 21. It is estimated that 1 in 700 newborn babies in United States are diagnosed with DS [1]. The phenotypes of DS frequently include congenital heart disease, intellectual disability, developmental abnormity and dysmorphic features [2]. Despite the fact that DS is mainly caused by trisomy 21, the genotype-phenotype association of typical DS features is yet to be determined.

Down Syndrome Critical Region (DSCR) hypothesis failed to provide solid evidence on the proposed theory of minimum gene responsible for all major DS phenotypes caused by a gene dosage effect [3–6]. Meanwhile, under limited circumstances partial monosomy 21 and partial trisomy 21 have been found to provide better understanding on the genotype-phenotype association of chromosome 21 [7–10]. Phenotypes with partial duplication or deletion of chromosome 21q are found to be highly variable among patients For instance, a child with Intellectual disability and dysmorphologic features of DS but without congenital heart disease was found to have a 2.78-Mb duplication on chromosome 21q22.11 [11]. Partial deletion of 21q21.1 are found to be associated with intellectual disability while deletion of 21q22.11 are considered associated with neurobehavioral disorder [8]. Despite the efforts to link DS clinical features with genes and regions, the association map resolution is low and details still remain incomplete.

Instead of trying to find a DSCR, our study focused on finding specific genotype-phenotype associations by investigating rare patients which involved a partial CNV on chromosome 21. Advancements in technology in comparative genomic hybridization (CGH) microarray enable laboratory to identify copy number variations (CNVs) on chromosome 21 as small as 10 k base pairs. From 2008 to 2018, 2768 samples were collected at the Genetics Laboratory at University of Oklahoma Health Sciences Center (OUHSC). During this period, we identified six patients with partial duplication and seven patients with partial deletion of chromosome 21. Among 13 patients with CNVs, two patients are related while others are independent. In this study, we report the molecular and clinical relationship of chromosomal imbalance on chromosome 21 and compare our results with current public data and literature to provide new way to naming patient with certain phenotypes.

## Methods

### Patients

The study was approved by the Institutional Review Board (IRB) of the University of Oklahoma. IRB number was 5938 and the reference number was 670,840. Retrospectively, 2768 pediatric patients sample were collected from 2008 to 2018 at the Genetics Laboratory at OUHSC. In-house software was developed to extract CNVs based on the following criteria:

1. CNVs only on chromosome 21, exclude whole chromosome 21 duplication/deletion and concurrent CNVs on other chromosomes.
2. CNV length larger than 100 kb.
3. CNV mean log2 ratio absolute value larger than 0.3.
4. CNV not overlap with common CNVs and segmental duplication regions.

After filtration, CNVs were manually curated to eliminate false positive cases based on background signal noise. A total of 13 samples were identified, six samples displayed partial duplication and seven samples showed partial deletion. The clinical information of the patients is summarized in Table 1.

**Table 1:** Molecular profile of patients

Patient #	Sex	Age at study	Chr21 location (hg19)	Gain/Loss	Size of CNVs (Mb)	Clinical features
DECIPHER280573	F	14y	18,582,895-18,983,265	Gain	0.4	DD, Intrauterine growth retardation, microcephaly, alopecia
S4	F	32 y	18,776,205-19,072,251	Gain	0.3	DD, Mother of S3
S1	M	2 y	18,781,100-18,885,813	Gain	0.1	DD, cleft palate, microcephaly, failure to thrive
S3	F	7 y	18,781,100-19,071,857	Gain	0.3	DD, pulmonary stenosis, autism, mild dysmorphic, daughter of S4
DECIPHER273421	M	4y	18,791,730-19,136,039	Gain	0.3	Intellectual disability
DECIPHER301183	F	2y	18,819,200-19,036,035	Gain	0.2	DD, microcephaly, seizures, hearing and visual abnormality
DECIPHER257242	F	7y	18,888,629-18,983,265	Gain	0.1	DD, Intrauterine growth retardation, intellectual disability
D6	F	2 y	21,427,690-24,133,154	Loss	2.7	DD
D4	F	33 y	22,271,313-23,399,894	Loss	1.1	Mother with Intrauterine fetal demise
D5	F	4 y	23,793,678-23,948,945	Loss	0.1	DD, epilepsia partialis continua
D3	M	2 m	23,840,342-23,941,319	Loss	0.1	Congenital coronary anomaly
D7	M	6 y	24,289,038-24,396,084	Loss	0.1	Seizures
S2	M	3 y	28,212,197-29,423,946	Gain	1.2	Preauricular skin tag, eye pterygium, speech delay
D2	F	1 y	31,005,158-31,236,003	Loss	0.2	Facial dysmorphic features
S6	F	9 d	35,902,679-36,149,583	Gain	0.2	Diaphragmatic hernia
S5	M	4 y	37,474,069-37,611,689	Gain	0.1	Pain insensitivity and minor dysmorphisms
D1	M	10 y	37,540,692-39,328,135	Loss	1.7	Microcephaly, levocurvature of thoracolumbar spine
Yamamoto, 2011	F	13y	38,528,931-39,009,341	Loss	0.4	DD, seizures, mild brain atrophy
Courcet, 2012	F	4y	38,722,631-38,791,771	Loss	0.1	Microcephaly, DD, ataxic gait, seizures
DECIPHER258106	F	N/A	38,865,151-38,885,792	Loss	0.1	Microcephaly, scoliosis, deeply set eye, Intrauterine growth retardation, short nose, sparse scalp hair, Hypoglycemia, DD, seizures
Van Bon, 2011	F	>20y	38,874,630-38,927,130	Loss	0.1	Microcephaly, DD, mild brain atrophy, anxious and autistic behaviour

*Note*: *F* female, *M* male, *y* year, *m* month, *d* day, *DD* developmental delay

### CGH microarray

Fresh blood samples were collected for this study and genomic DNA was extracted from peripheral white blood cells according to our standard operating using Nucleic Acid Isolation System (QuickGene-610 L, FUJIFILM Corporation, Tokyo, Japan). Patient D1’s CGH microarray was performed on a 385-K oligonucleotide chip and all other samples were performed on a CGH 720 K Whole-Genome Tiling v3.0 array (Roche NimbleGen, Inc., Madison, WI) according to the manufacturer’s protocol with minor modifications. As an internal hybridization control for each experiment, an opposite sex DNA came from normal population individuals pooled use as reference DNA (Promega Corporation, Madison, WI). Both the patients’ DNA and reference DNA were labeled with either Cyanine 3 (Cy-3) or Cyanine 5 (Cy-5) by random priming (Trilink Biotechnologies, San Diego, CA) and then hybridized to the chip via incubation in the MAUI hybridization system (Biomicro Systems, Inc., Salt Lake City, UT). After 18 h of hybridization at 42 °C, slides were washed and scanned using an MS200 (Roche NimbleGen, Inc.). NimbleScan version 2.4 and the SignalMap version 1.9 were applied for data analysis (NimbleGen System Inc., Madison, WI). CGH microarray results were analyzed referring to University of California Santa Cruz (UCSC) Genome Browser (GRCh37/hg19) (http://genome.ucsc.edu/cgi-bin/hgGateway). Frequently affected regions that were recently identified as copy number polymorphisms were excluded from data analysis according to the Chromosome Number Variation (CNV) database in our lab and genomic variants in human genome (Build 37). Variants of interest were compared to disease-causing genes in DECIPHER v8.7 (Database of Chromosomal Imbalance and Phenotype in Humans using Ensembl Resources) (decipher.sanger.ac.uk/index), DGV (Database of Genomic Variants) (dgv.tcag.ca/dgv/app/home), ClinVar (www.ncbi.nlm.nih.gov/clinvar) and OMIM (www.omim.org). The patient’s clinical phenotype and variants of interest were compared with available information from published reports.

## Results

### Duplications

**Fig. 1:**
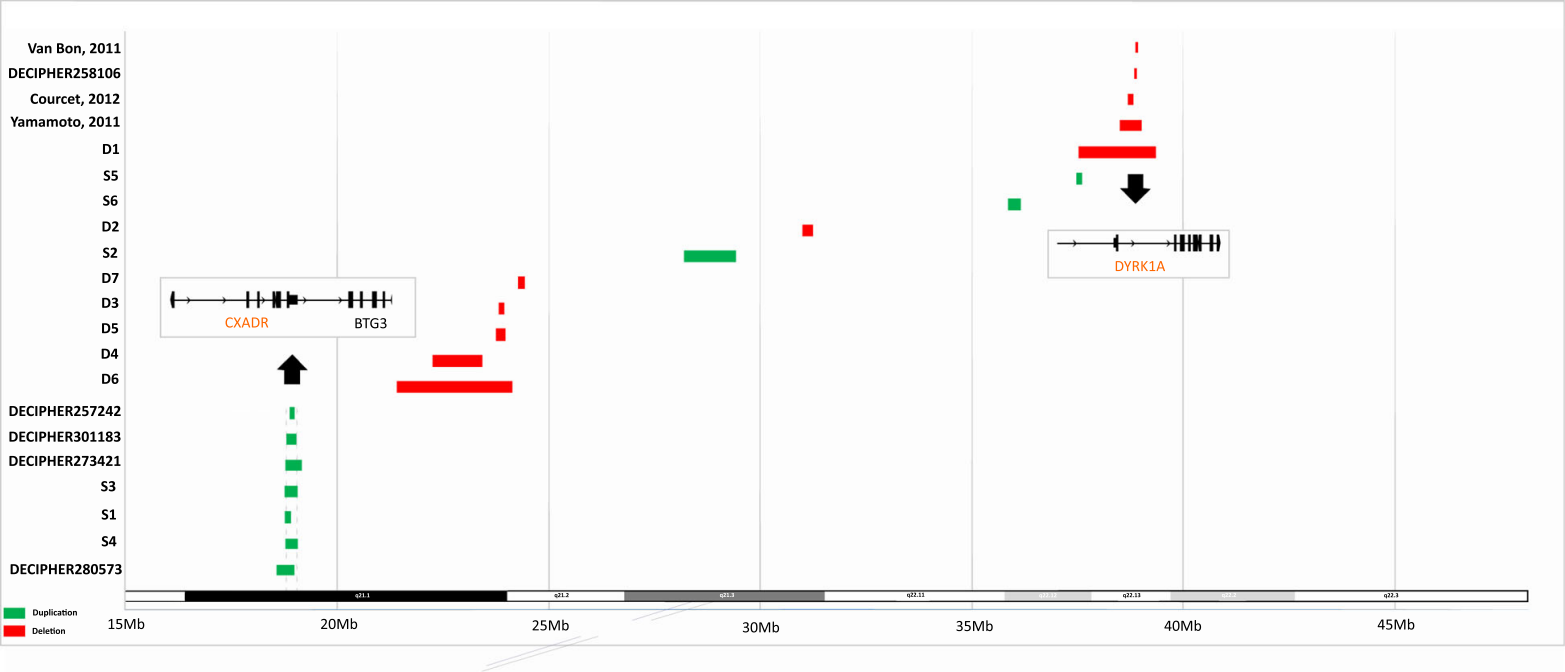
CNVs identified in this study and previous reported patients along chromosome 21. Note: Red color indicate copy number loss, green color indicate copy number gain. The order of the case follows Table 1

**Fig. 2:**
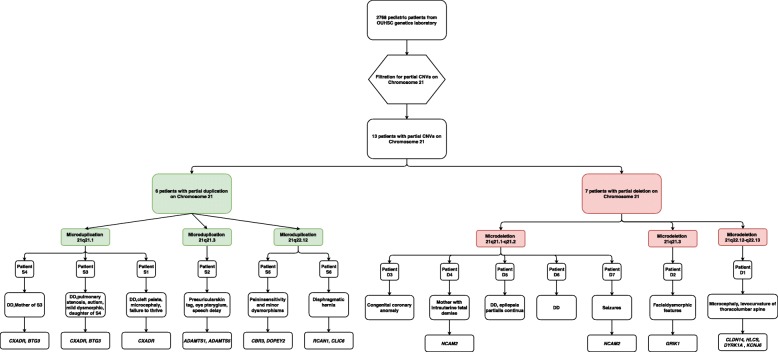
Flow chart of CNVs analysis and corresponding phenotype with OMIM genes. Note: Pink color indicate copy number loss, light green color indicate copy number gain. F = female, M = male, y = year, m = month, d = day, DD = developmental delay

Six patients (S1, S2, S3, S4, S5 and S6) were identified with duplication on chromosome 21. The size of chromosome 21 duplications ranged from 0.1 Mb to 1.2 Mb (Table 1, Fig. 1, and Additional file 1: Figure S1). Among the duplications, one CNV at 21q21.1 was maternally inherited by patient S3 from patient S4 and presented in another unrelated patient (S1) that includes the *CXADR* gene (Figs. 1 and 2). These three patients share the phenotype of developmental delay while patient S3 also demonstrated pulmonary stenosis and autism. A 1.2-Mb duplication at 21q21.3 presented in patient S2 with preauricular skin tag, eye pterygium and speech delay (Fig. 2). Two genes, *ADAMTS1* and *ADAMTS5,* have been identified on this CNV. Diaphragmatic hernia presented in patient S6 with a 0.2-Mb duplication at 21q22.12 which includes the *RCAN1* and *CLIC6* genes (Fig. 2). Also with duplication at 21q22.12, another patient (S5) showed pain insensitivity along with minor dysmorphism. Two protein-coding genes, *CBR3* and *DOPEY2,* are located in this duplication region (Fig. 2).

### Deletions

Seven patients (D1, D2, D3, D4, D5, D6 and D7) were identified with deletion on chromosome 21 ranged from 0.1 Mb to 2.7 Mb.Five patients (D3, D4, D5, D6 and D7) with various sizes of deletion at 21q21.1-q21.2 presented clinical features which included developmental delay, intrauterine fetal demise, epilepsia partialis continua, congenital coronary anomaly and seizures (Table 1, Figs. 1 and 2 and Additional file 1: Figure S1). The *NCAM2* gene is located in this 21q21.1-q21.2 deletion region (Figs. 1 and 2). Patient D2 had a phenotype of facial dysmorphic features and a 0.2-Mb deletion on 21q21.3 that includes the *GRIK1* gene (Figs. 1 and 2). Patient D1 presented with a deletion of 1.7 Mb at 21q22.12-q22.13 that includes four OMIM genes: *CLDN14*, *HLCS*, *DYRKIA* and *KCNJ6* (Figs. 1 and 2). The phenotype associated with this patient was microcephaly and levocurvature of the thoracolumbar spine.

## Discussion

### Microduplication 21q21.1

The microduplication on 21q21.1 was found in three patients S1, S3 and S4. This CNV was inherited from S4 to S3. Published reports of this CNV are rare but four patients (DECIPHER 257242, 301,183, 273,421 and 280,573) were found to have similar duplication segments and shared phenotypes such as developmental delay and intellectual disability [12]. There are two protein-coding genes, *CXADR* and *BTG3*, in this region. The CNV identified with patient S1 is within the 0.3-Mb region but is smaller and only includes the *CXADR* gene. Patient S1 also displayed the same developmental delay features that patients S3 and S4 possessed.

The *CXADR* and *BTG3* genes were found to be expressed during early brain development and a balanced expression of *BTG3* is critical for neuron differentiation in the forebrain [13, 14]. *BTG3* is upregulated in trisomic fibroblasts compared to a control indicating a gene dosage effect on heart tissues [15]. Considering patient S3 displayed pulmonary stenosis while patient S1 did not, and the extra duplicated region of S3 includes *BTG3*, this gene could potentially contribute to this clinical distinction. *BTG3* has also been linked with autism because of its role in cellular apoptosis and responses to redox changes [16, 17]. The protein product of *BTG3* has also been found to have a critical impact on cell growth and differentiation of other cells like T lymphocytes, fibroblasts and epithelial cells [18].

*CXADR* has been associated with developmental delay in two patients with larger CNV deletions of 7.9 Mb and 8.5 Mb [19, 20]. Combined with our three cases and the four DECIPHER patients mentioned above, it shows a strong connection between the irregular expression levels of *CXADR* and developmental disorders [19]. Coxsackievirus and adenovirus receptor (CAR) is a protein encoded by the *CXADR* gene. CAR has dual function as a receptor in the immune response against virus and a signal transduction molecule during neurological system development [21]. CAR is highly expressed in tissues like brain and heart in the early development; it is mainly expressed in endothelial cells and cardiac cells postnatally [22, 23]. Excessive expression of CAR has been link to activation of mitogen-activated protein kinase (MAPK) pathway in heart which might contribute to the hyper M1 inflammatory response in DS [24]. Additional follow up with Cardiologist might provide better understanding on the clinical significance of *CXADR* gene for these three patients. Our study narrowed down the critical region of patients with microduplication 21q21.1 features from 0.4 Mb to 0.1 Mb and highlighted the clinical relevance of *CXADR* gene as a potential cause for developmental delay, abnormal development of cardio myocytes and intellectual disability [25, 26].

### Microduplication 21q21.3

One patient S2 was found to have a 1.2-Mb microduplication at 21q21.3 with a preauricular skin tag, eye pterygium and speech delay. Our patient’s CNV is the first ever reported on this region which includes *ADAMTS1* and *ADAMTS5*. The functions of these genes are not well understood but *ADAMTS1* was found to be associated with various inflammatory processes and cachexia and *ADAMTS5* may involve destruction of the aggrecan, a cartilage proteoglycan [20]. One case of an 8.8-Mb deletion which encompasses both *ADAMTS1* and *ADAMTS5* was reported before with a similar speech delay phenotype as patient S2 but without other clinical features [20]. Further study is needed to better understand the clinical significance of this CNV.

### Microduplication 21q22.12

One patient S6, a 9-day-old female with a diaphragmatic hernia, was found to have a 0.2-Mb microduplication at 21q22.12. There are two genes in this region, *RCAN1* and *CLIC6*. Among them, *RCAN1* has been linked to contribute to the intellectual disability and neuronal degeneration in Alzheimer’s disease [27]. Multiple studies using animal models and transcriptome analysis demonstrated the important function by *RCAN1* in regulation of the anxiogenic response and oxidative stress-induced apoptosis [28–30]. Unfortunately, we were limited by the age of our patient (S6) so no other information is available other than the presence of the diaphragmatic hernia, which has not been found to be associated with either *RCAN1* or *CLIC6* before. The function of *CLIC6* is not exactly clear.

Another patient S5 was found to have a 0.1-Mb microduplication about 1.3 Mb downstream of S6’s CNV. Literature research found several patients had this CNV and it mostly resulted in nervous system disorders or developmental delay (DECIPHER 276835,287,879, 317,546 etc.) [12]. Interestingly, one case reported a 6.5-Mb deletion on 21q22.12 and the patient also showed signs of pain insensitivity, however the genotype is deletion instead of duplication, the overlap region coordinate is chr21: 37,474,069-37,554,434 (hg19) and *CBR3* gene is in this region [31]. Two genes, *CBR3* and *DOPEY2*, are found in the duplicated region of patient S5. These genes were both found to be closely related with DS phenotypes and be subject to the gene dosage effect causing intellectual disability and early onset of Alzheimer’s Disease [32–34]. However, no other pain insensitivity information was found to be associated with these genes.

### Microdeletion 21q21.1-q21.2

Five patients (D3, D4, D5, D6 and D7) were found with various sizes of deletion at 21q21.1-q21.2. Patient D6 had a 2.7-Mb microdeletion on 21q21.1-q21.2. Three patients (D3, D4 and D5) also had microdeletions of 0.1 Mb, 1.1 Mb and 0.1 Mb, respectively, within the 2.7-Mb microdeletion region of patient D6. Patient D7 had a 0.1-Mb microdeletion on 21q21.2 with a clinical feature of seizures. A previous study showed one patient (DECIPHER 319386) had a similar, but slightly smaller, microdeletion compared to patient D6. DECIPHER 319386 had macrocephaly, autistic behavior and delayed speech and language development but no common phenotypic features were found between patient D6 and DECIPHER 319386. A common feature found in patients D5 and D6 was developmental delay. Patient D4 was a mother who experienced an intrauterine fetal demise. A congenital coronary anomaly was reported in D3.

On the molecular level, *NCAM2* overlaps with the microdeletion on patients D4 and D6. *NCAM2* is believed to be associated with certain DS phenotypes because, as the expression levels change, multiple folds related to the homotypic adhesion properties of cells will be altered [35]. *NCAM2* also has been suggested as a candidate gene for autism because it is highly expressed in the brain and nervous systems [20, 36–38]. There are no known protein-coding genes in the microdeletion region of patients D3, D5 and D7 which suggests an alternative explanation other than CNVs is responsible for their phenotype.

### Microdeletion 21q21.3

One patient D2 had a microdeletion on 21q21.3 the size of 0.2 Mb and was found to be within the 0.4-Mb microdeletion region of DECIPHER 257308. Limited by the young age of patient D2 (one-year-old), only facial dysmorphic features were observed while DECIPHER 257308 displayed aggressive behavior, generalized tonic-clonic seizures and neurological speech impairment. *GRIK1*, a gene that encodes for a glutamatergic receptor subunit, is found in this microdeletion region. Glutamate is the most widely distributed excitatory neurotransmitter in the central nervous system acting on ionotropic and metabotropic receptors. Expression of *GRIK1* was found be to significantly lower in the hippocampus of DS patients and receptors were overexpressed in various areas of the brain [39, 40]. Also, trisomic animals were found to respond to glutamatergic stimuli differed from normal animals as well [41]. Based on the potential gene-dosage effect of *GRIK1* and the phenotypes displayed by DECIPHER 257308 which involved excitability, the *GRIK1* gene should be considered to be a strong candidate gene responsible for DS phenotypes.

### Microdeletion 21q22.12-q22.13

One patient D1 had a 1.7-Mb deletion on 21q22.12-q22.13, which includes four OMIM genes: *CLDN14, HLCS, DYRK1A* and *KCNJ6*. Yamamoto et al. (2011) reported two cases with mosaic deletions on 21q22qter and one case with a 0.4-Mb deletion within the 21q22.12-q22.13 microdeletion region. One of the mosaic deletion cases displayed an identical phenotype to patient D1 which included microcephaly and scoliosis [42]. In addition, a DECIPHER case (258106) that had a 0.2-Mb microdeletion within the same region also had microcephaly and scoliosis. *DYRK1A* is the only gene affected in all three cases and is reported to play a role in neurogenesis and neural differentiation [43]. Previous studies showed an association between *DYRK1A* and microcephaly had been well established and around half of the patients also displayed scoliosis [44, 45]. Single Nucleotide Variances and small INDELs on this genes also demonstrate similar phenotype of microdeletion on 21q22.13 that reaffirms this strong association between *DYRK1A* gene and syndrome with microcephaly and scoliosis [44, 45].

## Conclusions

In conclusion, our study expands the knowledge of the phenotypic consequences of CNVs on the long arm of chromosome 21. While the microduplications are associated with developmental delay, microcephaly, facial dysmorphic features, pulmonary stenosis, autism, preauricular skin tag, eye pterygium, speech delay and pain insensitivity, microdeletions are associated with developmental delay, microcephaly, intrauterine fetal demise, epilepsia partialis continua, congenital coronary anomaly and seizures. We suggest the *CXADR* gene is involved with developmental delay in patients with 21q21.1 microduplication and we provide additional evidence that *DYRK1A* is associated with microcephaly and scoliosis in patients with a 21q22 microdeletion. Both *CXADR* and *DYRK1A* are ranked high in the haploinsufficiency index of chromosome 21 gene list that indicates one allele with loss of function variant will result in a recognizable phenotype [46]. It also been found that haploinsufficient genes are more sensitive to dosage effect that might contribute to some of the DS phenotypes [47]. Our study demonstrates the clinical relevance of small CNVs as low as 0.1 Mb during CGH Microarray diagnostic testing and underlines the importance of prudent clinical interpretation of these CNVs. With the Next-generation sequencing (NGS) technology ability to detecting both single nucleotide variants and copy number variation in one test, Whole Genome Sequencing (WGS) could one day serve a more importance role in further establish the genotype-phenotype association. Large cohort studies with specific phenotypes subgroups will also be helpful to further our understanding of the genotype-phenotype association on chromosome 21.
